# Supplementary material for: Transparent flexible thermoelectric material based on non-toxic earth-abundant p-type copper iodide thin film
Source: Nat Commun. 2017 Jul 6;8:16076. doi: 10.1038/ncomms16076 (PMC5504294; doi:10.1038/ncomms16076)
Supplement: Supplementary Information — Supplementary Figures, Supplementary Table and Supplementary Notes [file ncomms16076-s1.pdf]

Title of file for HTML: Supplementary Information
Description: Supplementary Figures, Supplementary Table and Supplementary Notes

Title of file for HTML: Peer Review File
Description:

Supplementary Note 1. Structural properties and chemical composition

The crystal structure of obtained CuI thin film was determined by X-ray diffraction (XRD) using a Philips X'Pert x-ray diffractometer equipped with a Bragg-Brentano powder goniometer using divergent/ focusing slit optics and Cu Kα radiation. Supplementary Fig. 1a shows XRD 2θ-ω-scan of the CuI thin film grown on glass. The diffraction peaks correspond to the (111), (222), (333) planes of CuI in a zincblende structure, suggesting the growth of γ-phase CuI is along the [111] direction. No second phases have been detected.

The scanning electron microscopy (SEM) with Energy Dispersive X-Ray Analysis (EDX) was employed (FEI NOVA Nanolab 200) for examining the surface morphology and chemical composition of the films. As shown in Supplementary Fig. 1b, the SEM image clearly reveals triangularly shaped grains on glass substrate, which confirms the (111)-oriented γ-CuI according to XRD patterns. The random orientation of these grains indicates a thin film without in-plane epitaxial order. Hence, we conclude that the obtained CuI thin film has a (111) texture. These results are consistent with our previous reports[15-17].

The atomic ratio of Cu to I in the film determined by EDX was Cu:I = 1.1 ± 0.1:0.9 ± 0.1, indicating that the γ-CuI thin film was almost stoichiometric or slightly iodine-deficient. Hence, appropriately introducing more iodine into γ-CuI thin film would be able to increase the hole concentration.

Supplementary Note 2. Improvement of thermal stability

In order to improve the thermal stability of γ-CuI thin film, a 30-nm thick CuO_x layer was employed as a capping layer for protecting the CuI sample surface. Such an oxide capping layer could act as a barrier to prevent the iodine diffusion during thermal annealing. Here, the CuO_x layer was deposited by DC sputtering at room temperature using a high-purity (99.999%) copper target. The total amount of gas mixture was fixed to be 100 sccm, and the argon-to-oxygen ratio was 5:95. The as-deposited CuO_x layer was amorphous and insulating. Subsequently, the CuI thin films with and without a CuO_x capping layer were annealed in air at various temperatures for 5 min. The sheet resistance R_{sheet} of these samples are shown in Supplementary Fig. 3. It shows a three times reduced effect of increasing resistance and thus proves that further technological work targeted towards capping can extend the range of useful operation temperatures.

Supplementary Note 3. Evaluation of the maximum power output

For a thermoelectric module, the open circuit voltage V_{oc} is described by the equation $V_{oc} = S\Delta T$, where S is the Seebeck coefficient, and ΔT is the temperature difference. Hence, the power output P_{out} can be calculated using the equation $P_{out} = (S\Delta T)^2 R_{load}/(R_{load} + R_{int})^2$, where R_{load} is the load resistance, and R_{int} is the internal resistance of the thermoelectric module. Hence, the maximum power output P_{max} can be obtained when $R_{load} = R_{int}$, and described by $P_{max} = (S\Delta T)^2/4R_{int}$[6,28]. Accordingly, the P_{max} of the obtained single-leg module in this work can be evaluated, and the maximum power density is shown in Supplementary Fig. 4.

Supplementary Note 4. Evaluation of the Energy conversion efficiency

The ideal absolute maximum efficiency of a thermoelectric power generator is Carnot efficiency, given by $\eta_{carnot} = 1 - T_c/T_h$, where T_c is the temperature of cold side, and T_h is the temperature of hot side. The maximum conversion efficiency of a single-leg thermoelectric power generator can be calculated using $\eta = \eta_{carnot} [(1 + ZT)^{1/2} - 1]/[(1+ZT)^{1/2} + T_c/T_h]$, as shown in Supplementary Fig. 5.

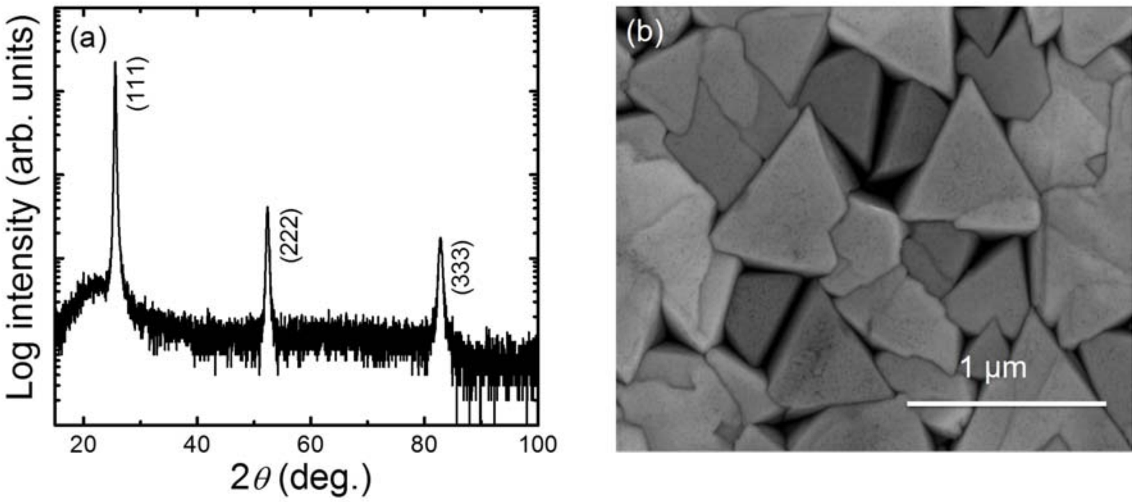

Supplementary Figure 1. Structural properties of the CuI thin films.

(a) XRD 2θ-ω-scan and (b) top-view SEM image of CuI thin film grown on glass.

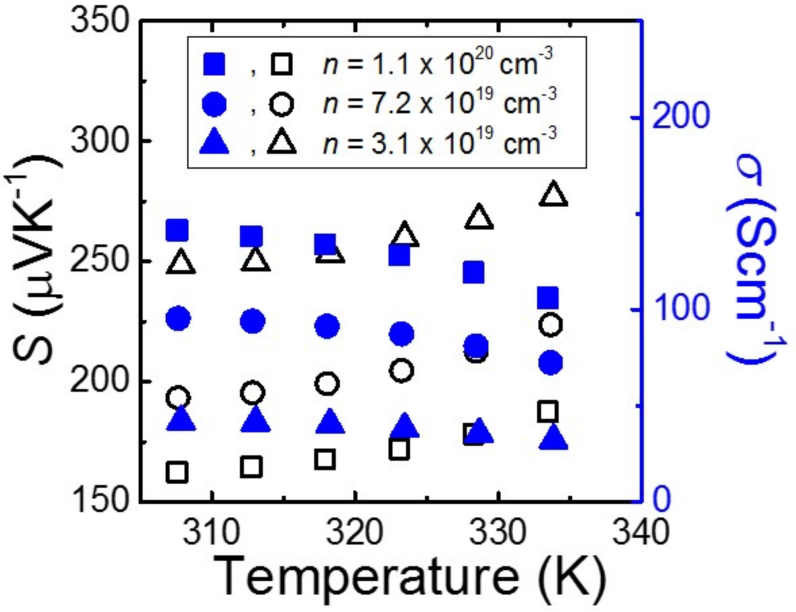

Supplementary Figure 2. Temperature dependence of thermoelectric transport properties. Seebeck coefficients S and electrical conductivity σ of CuI thin films deposited on glass at slightly elevated temperatures .

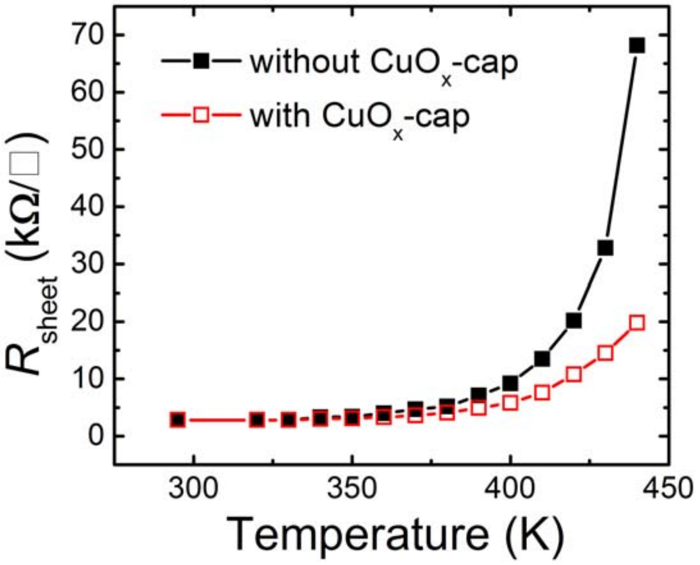

Supplementary Figure 3. Thermal stability of CuI thin films.

Sheet resistance R_{sheet} of CuI thin films with and without a CuO_x capping layer after annealing in air at various temperatures.

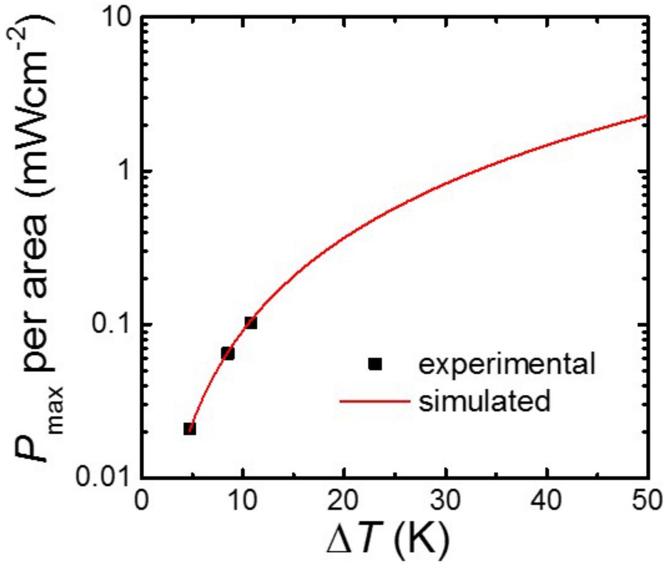

Supplementary Figure 4. Power density of CuI-based thermoelectric module.

Maximum power densities for various temperature differences.

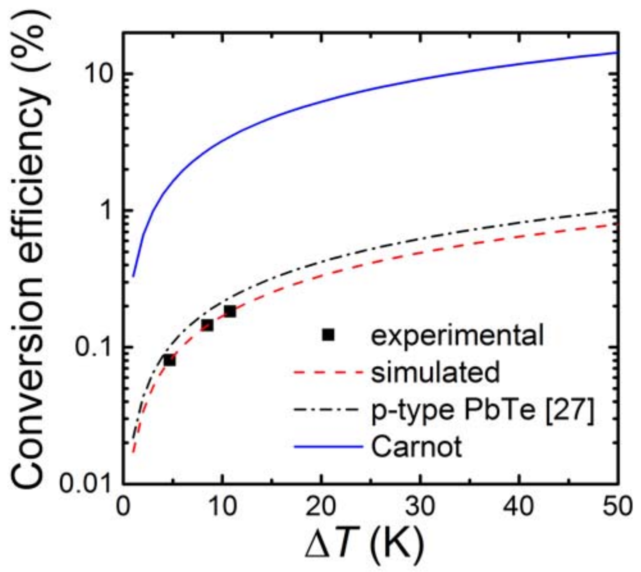

Supplementary Figure 5. Energy conversion efficiency.

The maximum conversion efficiency of a single-leg thermoelectric power generator based on CuI and PbTe for different temperature differences.

Supplementary Table 1. Physical properties of various thermoelectric materials.

Carrier type, energy bandgap and room-temperature thermoelectric performance for the CuI thin film and other typical thermoelectric materials.

**Carrier type**	**Material**	**Bandgap (eV)**	$S^2\sigma$ **$(10^{-3}Wm^{-1}K^{2})$**	κ **$(Wm^{-1}K^{-})$**	***ZT* at 300 K**	**Reference**
n	Bi_2Te_3	0.21	4.84	1.3	1.1	36
	$LaSrTiO_3$	2.3 – 3.2	2.3	9.1	0.08	11, 37
	Al:ZnO	3.4	0.39	1.19	0.1	9
	In:SnO_2	> 3.5	1.8	4	0.14	10
p	PbTe	0.32	3	3	0.3	28
	CuSe	1.23	0.6 – 0.8	0.8	0.2	19
	$Ca_3Co_4O_9$	2.1	0.83	3.5	0.07	14
	$CuAlO_2$	3.5	0.01	3.5	0.001	12
	$CuCrO_2$	2.5-3.0	0.06	7.5	0.002	13
	γ-CuI	3.1	0.375	0.5	0.21	*this work*
